# Midpoint of sleep is associated with sleep quality in older adults with and without symptomatic Alzheimer’s disease

**DOI:** 10.1093/sleepadvances/zpae023

**Published:** 2024-04-15

**Authors:** Scott C Sauers, Cristina D Toedebusch, Rachel Richardson, Adam P Spira, John C Morris, David M Holtzman, Brendan P Lucey

**Affiliations:** Department of Neurology, Washington University School of Medicine, St Louis, MO, USA; Department of Neurology, Washington University School of Medicine, St Louis, MO, USA; Department of Neurology, Washington University School of Medicine, St Louis, MO, USA; Department of Mental Health, The Johns Hopkins Bloomberg School of Public Health, Baltimore, MD, USA; Department of Psychiatry and Behavioral Sciences, The Johns Hopkins School of Medicine, Baltimore, MD, USA; The Johns Hopkins Center on Aging and Health, Baltimore, MD, USA; Department of Neurology, Washington University School of Medicine, St Louis, MO, USA; Knight Alzheimer Disease Research Center, Washington University School of Medicine, St Louis, MO, USA; Department of Neurology, Washington University School of Medicine, St Louis, MO, USA; Knight Alzheimer Disease Research Center, Washington University School of Medicine, St Louis, MO, USA; Center on Biological Rhythms and Sleep, Washington University School of Medicine, St Louis, MO, USA; Hope Center for Neurological Disorders, Washington University School of Medicine, St Louis, MO, USA; Department of Neurology, Washington University School of Medicine, St Louis, MO, USA; Center on Biological Rhythms and Sleep, Washington University School of Medicine, St Louis, MO, USA; Hope Center for Neurological Disorders, Washington University School of Medicine, St Louis, MO, USA

**Keywords:** aging, neurodegenerative disorders, REM sleep, slow-wave sleep

## Abstract

**Introduction:**

Disrupted sleep is common in individuals with Alzheimer’s disease (AD) and may be a marker for AD risk. The timing of sleep affects sleep–wake activity and is also associated with AD, but little is known about links between sleep architecture and the midpoint of sleep in older adults. In this study, we tested if the midpoint of sleep is associated with different measures of sleep architecture, AD biomarkers, and cognitive status among older adults with and without symptomatic AD.

**Methods:**

Participants (*N* = 243) with a mean age of 74 underwent standardized cognitive assessments, measurement of CSF AD biomarkers, and sleep monitoring via single-channel EEG, actigraphy, a home sleep apnea test, and self-reported sleep logs. The midpoint of sleep was defined by actigraphy.

**Results:**

A later midpoint of sleep was associated with African-American race and greater night-to-night variability in the sleep midpoint. After adjusting for multiple potential confounding factors, a later sleep midpoint was associated with longer rapid-eye movement (REM) onset latency, decreased REM sleep time, more actigraphic awakenings at night, and higher < 2 Hz non-REM slow-wave activity.

**Conclusions:**

Noninvasive in vivo markers of brain function, such as sleep, are needed to track both future risk of cognitive impairment and response to interventions in older adults at risk for AD. Sleep timing is associated with multiple other sleep measures and may affect their utility as markers of AD. The midpoint of sleep may be changed through behavioral intervention and should be taken into account when using sleep as a marker for AD risk.

**Statement of Significance**

The timing of sleep affects sleep-wake activity and is also associated with Alzheimer’s disease (AD). Little is known about links between sleep and the midpoint of sleep in older adults. In this study, we tested if different measures of sleep and the sleep midpoint are associated among older adults with and without symptomatic AD. Older adults (N=243) underwent standardized cognitive assessments, measurement of AD biomarkers, and at-home sleep monitoring. A later midpoint of sleep was associated with longer REM onset latency, decreased REM sleep time, and increased <2 Hz non-REM slow-wave activity. Sleep timing is associated with multiple sleep measures and should also be taken into account when using sleep as a marker for AD risk.

Alzheimer’s disease (AD) is a progressive neurodegenerative disease characterized neuropathologically by insoluble extracellular plaques of amyloid-β (Aβ) and intracellular neurofibrillary tangles of hyperphosphorylated tau (p-tau) resulting in cognitive decline, dementia, and death [1]. AD is a growing public health crisis with the global prevalence of AD and other dementias expected to increase from 57 million cases as of 2019 to 153 million cases in 2050 [2]. Amyloid pathology emerges ~15–20 years before neuronal loss and subsequent cognitive decline [3]. Although there are many markers for AD pathology including the soluble cerebrospinal fluid (CSF) concentration of Aβ42 [4], the CSF and plasma Aβ42/40 ratio [5, 6], the levels of CSF and plasma p-tau species [7, 8], and radiotracers that bind to Aβ and tau via positron emission tomography [9–11], a major goal of the field is to develop AD biomarkers of brain function to identify individuals who are at risk for cognitive impairment before symptom onset. Sleep is a potential noninvasive in vivo marker of brain function that could be followed to track future risk of cognitive impairment as well as response to interventions such as anti-amyloid monoclonal antibodies.

Multiple measures of sleep and sleep disorders have been associated with an increased risk of AD or cognitive decline including poor self-reported sleep quality [12], short or long sleep durations [13], fragmented sleep [14], decreased sleep efficiency [15], increased wake after sleep onset [16], increased sleep onset latency [17], increased rapid-eye movement (REM) sleep onset latency [18], time spent in different sleep stages [19, 20], sleep-disordered breathing [21], and periodic limb movement during sleep [22]. Sleep and AD are hypothesized to have a bi-directional relationship with disrupted sleep contributing to the development of AD and AD pathology resulting in sleep disturbances [23, 24]. Disrupted sleep behavior also is associated with increased risk of AD and future risk of cognitive impairment. For instance, daytime napping is linked to greater likelihood of amyloid pathology and future cognitive impairment even in cognitively unimpaired older adults [25–27].

Sleep timing is related to the body’s natural time to sleep, as well as an individual’s chronotype such as morningness or eveningness [28]. The midpoint of sleep or the midpoint of time in bed may be measured by actigraphy, sleep logs, or questionnaires and has been proposed as a measure of chronotype [29–32] although it is a measure of sleep behavior rather than the endogenous circadian rhythm. Individuals with both early and late midpoint of sleep are at risk for worse health outcomes, including increased risk for dementia [33–36]. Mendelian randomization analyses suggest a causal role of the later midpoint of sleep determined by actigraphy or questionnaires and increasing prostate cancer [37], poor mental health [38], and educational attainment [39], as well as a role of cognitive function in causing a later sleep midpoint [40]. Individuals with early and late midpoints of sleep are more at risk for spinal, gastrointestinal, respiratory, and cardiovascular diseases, as well as depression, anxiety, personality disorders, substance use disorders, infertility, obesity, diabetes, insomnia, sleep apnea, and all-cause mortality [41].

The potential to use sleep measures as a marker for AD risk is likely to be affected by timing of sleep given the overlapping relationships among sleep quality and disturbance, the timing of sleep (such as the sleep midpoint and daytime napping), and AD. In this study, we tested if different measures of sleep and the midpoint of sleep are related even after adjusting for multiple confounding variables such as age, sex, race, AD biomarkers, and cognitive status. We hypothesized that late sleep timing will be associated with measures of poorer sleep quality (e.g. less REM sleep). If sleep measures are affected by sleep timing, then sleep timing may need to be taken into account when using sleep as a marker for AD risk.

## Materials and Methods

### Participants

Data were gathered from 388 community-living participants enrolled in an ongoing longitudinal study of aging and AD (Healthy Aging and Senile Dementia) at the Knight Alzheimer Disease Research Center, Washington University in St. Louis. All individuals participating in Knight Alzheimer Disease Research Center studies undergo annual standardized clinical and cognitive assessments. The Clinical Dementia Rating® (CDR®) was used to determine if participants were cognitively unimpaired (CDR 0), or mildly impaired (CDR 0.5) at the time of referral. Participants were excluded if they had any psychiatric or systemic medical illness that can contribute to dementia. This study was approved by the Washington University in St. Louis Institutional Review Board. Each participant provided signed informed consent and was compensated for their participation.

### Sleep monitoring

Sleep was recorded longitudinally at home for up to 6 consecutive nights using self-reported sleep logs, actigraphy (Actiwatch2, Philips Respironics), and a single-channel EEG device worn on the forehead (Sleep Profiler, Advanced Brain Monitoring). The mean number of nights of actigraphy recordings used to derive sleep midpoint was 5.8. Sleep parameters were determined for the single-channel EEG, actigraphy, and sleep logs. Sleep-disordered breathing and periodic leg movements were measured using a home sleep apnea test (HSAT) device (Alice PDx, Philips Respironics), a device that was found to have 96.4% agreement with simultaneously recorded in-laboratory polysomnography [42].

#### Single-channel EEG.

Average total sleep time, time in non-REM (NREM) sleep stages 2 and 3 (time in NREM), time in REM sleep, sleep efficiency, and NREM slow-wave activity (SWA) were measured as previously described [43]. Sleep efficiency was calculated based on the lights off and lights on times for the single-channel EEG studies and which were corroborated with actigraphy and sleep logs. Single-channel EEG sleep studies were visually scored by registered polysomnographic technologists using criteria adapted from the standard American Academy of Sleep Medicine criteria [43]. Nights were excluded if > 10% of the recording was artifactual and if the bed and rise times did not match the sleep log and/or actigraphy. All participants needed at least 2 nights of single-channel EEG monitoring that met these criteria to be included in the analysis. Time in NREM sleep stages 2 and 3 were combined, as the combined metric has a higher level of agreement with polysomnography [20].

NREM SWA was calculated for each single-channel EEG study using MATLAB (MathWorks, Natick, MA), and the average NREM SWA in different frequency bins were used in the analysis. As previously described, a band-pass (two-way least-squares finite impulse response) filter between 0.5 and 40 Hz was applied to the single-channel EEG data. Spectral analysis was performed in consecutive 5-second epochs (Welch method, Hamming window, no overlap) [20]. SWA power was calculated by averaging the power in the frequency bins of 0.5–1.0, 1.0–2.0, 2.0–3.0, 3.0–4.0, and 1–4.5 Hz. To semi-automatically remove artifactual epochs, power in the 20–30 and 0.5–4.5 Hz bands for each electrode across all epochs of a recording were displayed. The operator (B.P.L.) then selected a threshold between the 95% and 99.5% threshold of power to remove artifactual epochs.

To determine NREM SWA dissipation as a measure of sleep homeostasis, we calculated the percent change in 1–4.5 Hz NREM SWA from the first to last 20 minutes of sleep calculated by subtracting the NREM SWA in the last 20 minutes of sleep from the SWA in the first 20 minutes of sleep, then dividing by the NREM SWA in the first 20 minutes of sleep. Greater negative percent change in 1–4.5 Hz NREM SWA indicates greater decline in NREM SWA between the first and last 20 minutes of sleep.

#### Actigraphy.

Actigraphy is commonly used to assess sleep and is validated against polysomnography [44]. Start and end times were set using a standardized protocol involving event marker button presses and sleep logs as previously described [45]. The epoch length was 30 seconds. The wake threshold selection was set to low with the wake threshold value set to 20. Sleep interval detection was based on immobile minutes. Both the sleep onset and sleep end settings were set to 10 minutes. Actigraphic sleep parameters were calculated using Actiware 6.0 (Philips Respironics) and included: total sleep time, sleep efficiency, sleep onset latency, wake after sleep onset, and number of awakenings.

#### Sleep logs.

Participants completed a sleep log for all nights that the single-channel EEG, actigraphy, and HSAT were worn. Self-reported sleep parameters included total sleep time, sleep onset latency, the number of awakenings during the night, and minutes of napping during the day.

#### Home sleep apnea test.

Participants were monitored at home for one night to measure sleep-disordered breathing and periodic leg movements. The Alice PDx is a type III HSAT device that monitors oxygen saturation (SpO2) and pulse rate from an oximeter finger probe, nasal pressure-based airflow monitor and thermistor, thoracic and abdominal effort via inductance plethysmography, bilateral electromyography sensors over the anterior tibialis muscles, and body position. Participants pressed the event button to monitor at lights off and lights on. Bed and rise times were also confirmed with sleep logs and actigraphy as previously described [45]. In the morning, participants checked the “good study” indicator on the device to confirm a minimum of 4 hours of recording. A minimum of 4 hours of artifact-free recording was obtained for all participants and participants not meeting this criterion were asked to repeat monitoring. Respiratory events and periodic leg movements were scored by registered polysomnographic technologists using American Academy of Sleep Medicine criteria [46] and were reviewed by a board-certified sleep medicine physician (B.P.L.). The criteria for scoring hypopneas was a 4% decrease in oxygen saturation. The calculation of the apnea–hypopnea index (AHI) and periodic leg movement index (PLMI) were calculated per hour of monitoring time for each participant. Participants using PAP therapy or dental devices were asked to use them as usual during the HSAT.

### Cerebrospinal fluid

CSF was collected under a standardized protocol [47]. After overnight fasting, participants underwent a lumbar puncture at 08:00 am and 20–30 mL of CSF was collected by gravity drip into a 50-mL conical tube using a 22-gauge atraumatic Sprotte spinal needle. The conical tube was then gently inverted to disrupt potential gradient effects and centrifuged at low speed to pellet any cellular debris. Samples were aliquoted (500 μL) in polypropylene tubes and stored at −80°C until analysis. CSF Aβ40 and Aβ42, total tau (t-tau), and phosphorylated tau-181 were measured as previously described using an automated electrochemiluminescence immunoassay (Lumipulse, Fujirebio) [48].

### Midpoint of sleep

A participant’s sleep timing was defined as the average midpoint of the participant’s sleep as measured by actigraphy. Actigraphy has previously been used to determine the midpoint of sleep [30, 31]. The midpoint of sleep was calculated for individual nights based on bedtimes and rise times, and then averaged for each participant. Midnight was set at “0” and times before midnight were negative (e.g. 11:00 pm = −1) and times after midnight were positive (e.g. 01:00 am = + 1). Participants were divided into equal groups of “early” and “late” sleep timing depending on whether they had an average midpoint of sleep before or after the average for the sample (03:04 am). Forty-three participants also completed the Munich Chronotype Questionnaire (MCTQ) [49] which collects information on sleep–wake schedules during work and free days. The MCTQ is used to determine the midpoint of sleep. Comparison of the midpoint of sleep determined by actigraphy and the MCTQ resulted in a high correlation (Supplementary Figure S1: *r* = 0.823; *p* = 1.278 × 10^−11^). Sleep timing variability was also calculated as the standard deviation of an individual’s sleep midpoints across multiple nights of monitoring. Higher sleep timing variability indicates greater night-to-night differences in the midpoint of sleep. Employment data were collected on average less than 4 months from the sleep study day and were available for 98% of the participants. Sleep midpoint was not correlated with employment (*p* = .48). Sleep midpoint was not adjusted for sleep duration, weekend days, or for the 26.5% of participants who reported employment.

### Statistical analysis

Differences between sleep timing groups were tested using Welch’s two-tailed *t*-test, or Chi-square if the data were categorical. Linear regression models tested if sleep timing predicted different sleep variables after adjusting for age, sex, race, CDR, APOE4 status, CSF Aβ42/40, AHI, PLMI, and self-reported minutes spent daytime napping. Only participants with data for age, sex, race, CDR, APOE4 status, CSF Aβ42/40, AHI, and PLMI were included in the main analyses. Coding for dichotomous variables in the regression model is as follows: 1 = male, 2 = female; 0 = non-African American, 1 = African American (race determined by self-report); CDR negative (a score of 0) = 1, CDR positive (a non-zero score) = 2. All analyses were performed using R. Statistical significance was set at α = 0.05.

### Data availability

Data to support the findings of this study are available from the corresponding author upon reasonable request. All code associated with this analysis is freely available from the corresponding author upon reasonable request.

## Results

### Participant characteristics

**Figure 1.**
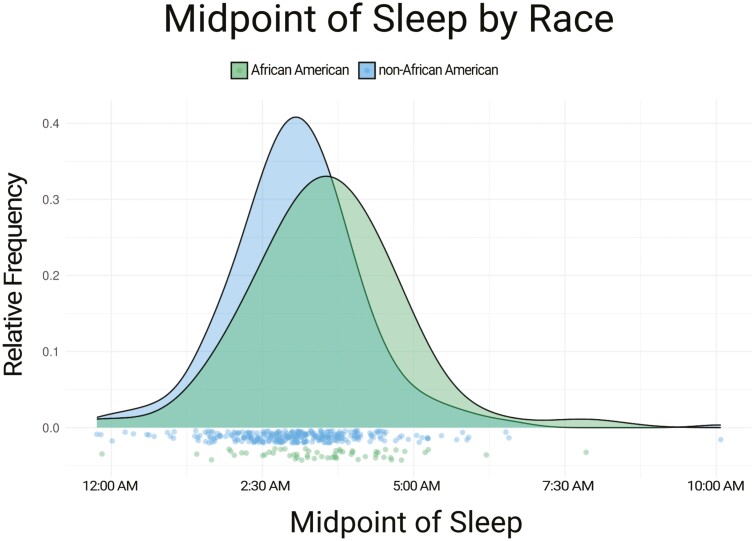
Distribution of midpoint of sleep by race. African Americans had a later midpoint of sleep than non-African Americans. For sleep midpoint, “0.0” is at midnight (12:00 am) and more positive numbers are later in the night (i.e. 5.0 is at 05:00 am). Green, African American; Blue, non-African American. Color version of the figure is available online.

Demographic and sleep parameter differences between the late and early midpoint of sleep groups are shown in Tables 1, 2, and 3. Sex and age were not significantly different between early and late midpoint sleep groups. African Americans (self-report black ethnic background) were significantly more likely to have a late sleep midpoint than non-African Americans and this relationship remained significant (*p* = .007) after including participants who were missing other data (and were not included in analyses using the fully adjusted model). This expanded sample included *N* = 58 African Americans and *N* = 293 non-African Americans. The distribution of sleep timing by race, including these additional participants, is shown in Figure 1. A total of 243 individuals were included in the main analyses because participants without data for actigraphic sleep midpoint, age, sex, race, CDR, APOE4 status, CSF Aβ42/40, AHI, and PLMI were excluded from the tables. Supplementary Figure S1 and Figure 1 do not exclude these participants.

**Table 1.** Participant Characteristics

	Early sleep timing	Late sleep timing	*P*-value
	Midpoint of sleep before 03:04 am (*n* = 122)	Midpoint of sleep after 03:04 am (*n* = 121)	
Age at sleep study, years, mean (SD)	73.785 (4.996)	73.589 (5.536)	.7717
Sex, number of females (%)	59 (48.361%)	68 (56.198%)	.2213
Race, number of African Americans, *n* (%)	7 (5.738%)	17 (14.05%)	**.0299**
Clinical Dementia Rating, *n* CDR + (% = 0.5)	25 (20.492%)	22 (18.182%)	.6485
APOE ε4 status: n APOE ε4 + (%)	45 (36.885%)	42 (34.711%)	.724
CSF Aβ42/Aβ40 ratio, mean (*SD*), and then *t*-test	0.0687 (0.0233)	0.0734 (0.0226)	.1754
Amyloid pathology, number Aβ+ (% CSF Aβ42/Aβ40 < 0.0673)	62 (50.82%)	51 (42.149%)	.1085
AHI, number of respiratory events/h of monitoring time (%)			.1798
None (< 5)	47 (38.525%)	60 (49.587%)	
Mild (5–<15)	49 (40.164%)	40 (33.058%)	
Moderate (15–<30)	20 (16.393%)	18 (14.876%)	
Severe (≥ 30)	6 (4.918%)	3 (2.479%)	
PLMI, number of leg movements/h of monitoring time (%)			.8073
None (< 15)	56 (45.902%)	65 (53.719%)	
Low (15–<45)	39 (31.967%)	30 (24.793%)	
High (≥ 45)	27 (22.131%)	26 (21.488%)	
Sleep timing variability, mean (*SD*)	0.526 (0.263)	0.658 (0.405)	**.00299**

SD, standard deviation; CDR, clinical dementia rating; ApoE4 status, apolipoprotein E ε4 positive; CSF, cerebrospinal fluid; Aβ, amyloid-beta; AHI, apnea–hypopnea index; PLMI, periodic limb movement index.

Bold *P*-values are significant at <0.05.

**Table 2.** EEG Sleep Parameters

	Early sleep timing	Late sleep timing	*P*-value
	Midpoint of sleep before 03:04 am (*n* = 122)	Midpoint of sleep after 03:04 am (*n* = 121)	
Total sleep time, min, mean (*SD*)	371.7555 (57.798)	377.9669 (66.287)	.4452
Sleep efficiency, %, mean (*SD*)	78.77617 (9.115)	78.24584 (9.871)	.6693
Sleep onset latency, min, mean (*SD*)	17.19489 (11.865)	19.06408 (13.855)	.2684
Wake after sleep onset, min, mean (*SD*)	141.076 (21.207)	144.8782 (29.037)	.2454
N2 and N3 time, min, mean (*SD*)	254.2989 (51.253)	270.4797 (63.122)	**.03231**
REM time, min, mean (*SD*)	81.13711 (25.163)	72.3852 (24.22)	**.007088**
REM latency, min, mean (*SD*)	99.99076 (62.856)	128.29124 (78.748)	**.002652**
NREM SWA < 1 Hz, μV2/Hz, mean (*SD*)	58.04261 (40.302)	75.09838 (60.766)	**.01248**
NREM SWA 1–2 Hz, μV2/Hz, mean (*SD*)	39.58187 (25.023)	49.44853 (33.736)	**.012**
NREM SWA 2–3 Hz, μV2/Hz, mean (*SD*)	12.01186 (6.869)	13.5532 (7.187)	.09507
NREM SWA 3–4 Hz, μV2/Hz, mean (*SD*)	5.332111 (2.825)	5.830836 (2.917)	.1855

Min, minutes; SD, standard deviation; N2, stage N2 sleep; N3, stage N3 sleep; REM, rapid-eye-movement sleep; NREM SWA, non-REM slow-wave activity; Hz, Hertz.

Bold *P*-values are significant at <0.05.

**Table 3.** Actigraphy and Self-Reported Sleep Parameters

	Early sleep timing	Late sleep timing	*P*-value
	Midpoint of sleep before 03:04 am (*n* = 122)	Midpoint of sleep after 03:04 am (*n* = 121)	
Actigraphic total sleep time, min, mean (*SD*)	390.8058 (51.996)	397.5094 (64.132)	.3719
Actigraphic sleep efficiency, %, mean (*SD*)	81.65731 (7.291)	80.40583 (9.184)	.2409
Actigraphic sleep onset latency, min, mean (*SD*)	19.73729 (15.659)	24.01861 (24.961)	.1122
Actigraphic wake after sleep onset, min, mean (*SD*)	149.4696 (21.075)	153.4047 (29.456)	.2327
Actigraphic number of awakenings, *n*, mean (*SD*)	32.65246 (12.499)	34.74959 (12.87)	.1988
Self-reported total sleep time, min, mean (*SD*)	427.47408 (62.1)	446.27718 (69.78)	**.03165**
Self-reported sleep onset latency, min, mean (*SD*)	25.52586 (23.949)	23.94115 (19.837)	.5857
Self-reported number of awakenings, *n*, mean (*SD*)	2.126496 (1.143)	2.086725 (1.471)	.8196
Self-reported daytime minutes napping, mean (*SD*)	73.785 (20.142)	73.589 (22.125)	.7717

Min, minutes; SD, standard deviation.

Bold *P*-values are significant at <0.05.

**Figure 2.**
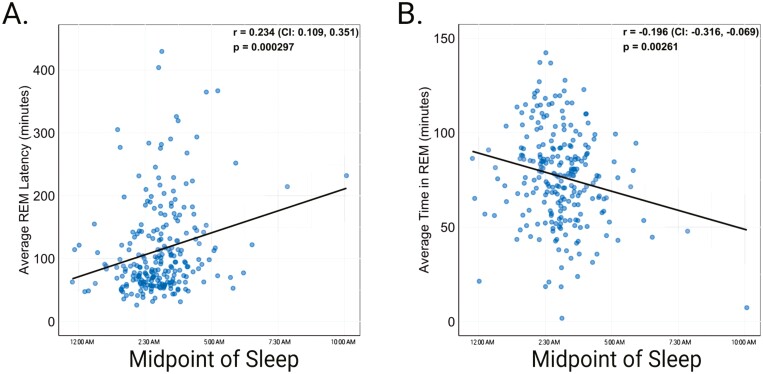
Relationship of the midpoint of sleep and REM sleep. As the midpoint of sleep became later in the night, average REM onset latency increased (A) and average time in REM sleep decreased (B). CI: confidence intervals.

**Figure 3.**
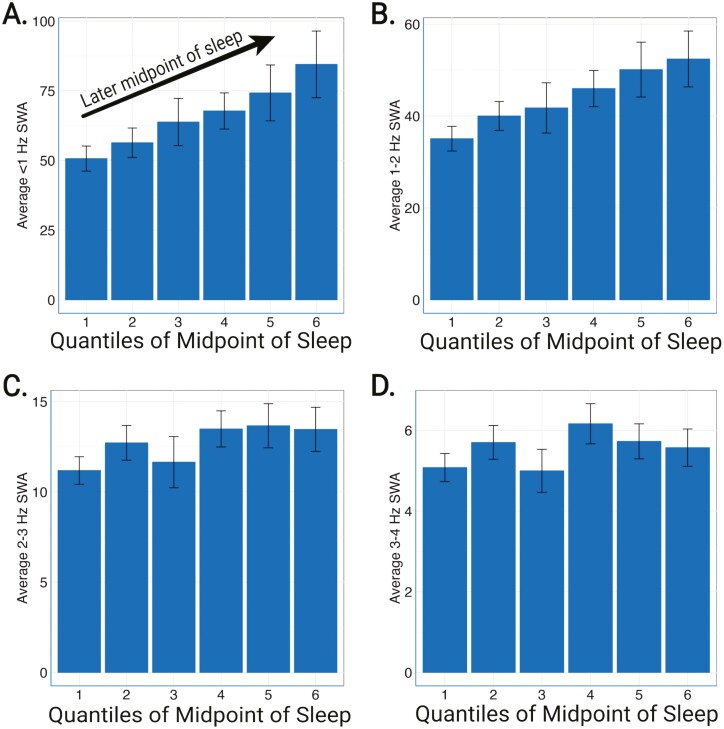
Relationship of the midpoint of sleep and NREM slow-wave activity. Rounded to the nearest minute, the midpoint of sleep was separated into quantiles of 11:46 pm to 02:07 am (first, leftmost), 02:07 am to 02:38 am (second), 02:38 am to 03:03 am (third), 03:03 am to 03:28 am (fourth), 03:28 am to 03:55 am (fifth), 03:55 am to 10:04 am (sixth, rightmost). < 1 Hz and 1–2 Hz NREM slow-wave activity (SWA) increased with later midpoint of sleep (A, B). The same relationship was not seen for 2–3 Hz NREM SWA (C) or 3–4 Hz (D).

Participants with later sleep timing had lower REM time, longer REM onset latency (Figure 2), and greater time in stages N2 and N3 (Table 2). Self-reported total sleep time, but not objective measures of sleep duration measured by either EEG or actigraphy, was significantly longer in those with later midpoints of sleep. < 1 Hz and 1–2 Hz NREM slow-wave activity were higher in participants with a later sleep midpoint (Figure 3; Table 2). Notably, individuals with later sleep timing also had significantly greater between-night sleep timing variability.

### EEG sleep parameters

Multiple EEG-based sleep parameters have been associated with cognitive deficits and AD pathology. However, sleep may be confounded by factors such as age, sex, ApoE4 + status, amyloid pathology, and others [50–53]. Therefore, we adjusted for age, sex, race, CDR, ApoE4 status, CSF Aβ42/40 ratio, AHI, PLMI, and self-reported time spent napping during the day to assess the relationship between EEG-derived sleep parameters and the midpoint of sleep (sleep timing). In the fully adjusted model, later sleep timing was associated with longer REM onset latency and shorter time in REM (Table 4). Longer REM onset latency was also associated with female sex and mild cognitive impairment; an interaction between midpoint of sleep and CDR was not significant (*p* > .05, data not shown). In contrast, biomarker evidence of amyloid pathology (lower Aβ42/40) was associated with shorter REM onset latency. Less time in REM was associated with greater age and self-reported black ethnic background. Sleep timing was not significantly associated with time in NREM stages 2 and 3, total sleep time, sleep efficiency, sleep onset latency, or wake after sleep onset (Table 4 and 5).

**Table 4.** EEG Sleep Stages

	Dependent variables					
	Time in N2 and N3 (min)		Time in REM (min)		REM Onset Latency (min)	
Covariates	Estimate (β)	*P*-value	Estimate (β)	*P*-value	Estimate (β)	*P*-value
Sleep timing	4.055	.186	−3.312	**.016**	11.498	**.002**
Age	0.699	.348	−0.750	**.025**	0.177	.842
Sex	16.507	**.026**	2.659	.420	43.241	**.001**
Race	−29.743	**.025**	−12.697	**.032**	−24.127	.127
CDR	8.908	.368	−3.446	.435	34.279	**.004**
ApoE4 status	10.541	.218	2.593	.497	19.719	.054
Aβ42/40	69.924	.703	−46.138	.573	546.868	**.013**
AHI	−1.372	**.001**	.091	.608	0.401	.400
PLMI	0.002	.992	−0.035	.608	0.356	.054
Daytime minutes napping	−0.090	.616	−0.085	.292	0.119	.581

Min, minutes; SD, standard deviation; N2, stage N2 sleep; N3, stage N3 sleep; REM, rapid-eye-movement sleep; CDR, clinical dementia rating; ApoE4 status, apolipoprotein E ε4 positive; Aβ, amyloid-beta; AHI, apnea–hypopnea index; PLMI, periodic limb movement index.

Bold *P*-values are significant at <0.05.

**Table 5.** EEG Sleep Parameters

	Dependent variables							
	TST (min)		Sleep efficiency (%)		SOL (min)		WASO (min)	
Covariates	Estimate (*β*)	*P*-value	Estimate (*β*)	*P*-value	Estimate (*β*)	*P*-value	Estimate (*β*)	*P*-value
Sleep timing	3.821	.245	−0.752	.155	0.644	.366	1.775	.069
Age	0.054	.946	−0.101	.433	−0.008	.965	0.154	.507
Sex	18.724	**.019**	0.027	.983	6.183	**<.001**	10.116	**<.001**
Race	−44.699	**.002**	−2.732	.232	−0.142	.963	−12.477	**.003**
CDR	10.806	.309	−0.556	.744	1.103	.631	−0.728	.812
ApoE4 status	8.638	.346	0.889	.546	−0.247	.901	3.752	.167
Aβ42/40	−87.079	.658	−14.890	.638	43.417	.309	18.918	.745
AHI	−0.620	.148	−0.171	**.014**	0.036	.695	−0.185	.139
PLMI	0.015	.927	0.001	.966	−0.020	.575	−0.005	.914
Daytime minutes napping	−0.252	.192	0.012	.700	0.039	.350	−0.033	.559

TST, total sleep time; SOL, sleep onset latency; WASO, wake after sleep onset; min, minutes; CDR, clinical dementia rating; ApoE4 status, apolipoprotein E ε4 positive; Aβ, amyloid-beta; AHI, apnea–hypopnea index; PLMI, periodic limb movement index.

Bold *P*-values are significant at <0.05.

### Non-REM slow-wave activity

The observed difference in NREM SWA between early and late sleep timing groups (Figure 3; Table 2) could be due to other factors such as age, sex, and amyloid pathology. We tested this in the fully adjusted model and found that later sleep timing was significantly associated with higher < 1 Hz and 1–2 Hz NREM SWA (Table 6). Female sex was associated with higher NREM SWA in all frequency ranges. As previously reported [54, 55], lower NREM SWA (< 1, 1–2, and 2–3 Hz) was significantly correlated with lower CSF Aβ42/40 ratio (a marker for amyloid deposition). African Americans also showed decreased < 3 Hz NREM SWA.

**Table 6.** NREM Slow-Wave Activity

	Dependent variables							
	<1 Hz		1–2 Hz		2–3 Hz		3–4 Hz	
Covariates	Estimate (β)	*P*-value	Estimate (β)	*P*-value	Estimate (β)	*P*-value	Estimate (β)	*P*-value
Sleep timing	8.173	**.002**	3.226	**.032**	−0.012	.974	−0.126	.409
Age	1.003	.114	0.388	.289	0.008	.929	0.013	.728
Sex	39.415	**<.001**	24.343	**<.001**	5.917	**<.001**	2.236	**<.001**
Race	−28.265	**.012**	−16.703	**.010**	−3.436	**.029**	−1.178	.074
CDR	6.645	.433	3.472	.477	0.525	.657	0.228	.645
ApoE4 status	12.227	.093	5.006	.231	0.564	.578	0.318	.454
Aβ42/40	587.444	**<.001**	333.449	**<.001**	54.496	**.013**	12.720	.163
AHI	0.496	.142	0.183	.348	−0.020	.668	−0.018	.359
PLMI	0.164	.210	0.120	.113	0.033	.075	0.009	.231
Daytime minutes napping	0.334	**.029**	0.186	**.035**	0.039	.068	0.016	.069

NREM, non-rapid-eye movement; CDR, clinical dementia rating; ApoE4 status, apolipoprotein E ε4 positive; Aβ, amyloid-beta; AHI, apnea–hypopnea index; PLMI, periodic limb movement index; Hz, Hertz.

Bold *P*-values are significant at <0.05.

Longer self-reported daytime napping was also associated with higher < 1 and 1–2 Hz NREM SWA, suggesting that the increase in NREM SWA was due to the homeostatic response to greater sleep need. To test this possibility, we assessed the dissipation of 1–4.5 Hz NREM SWA measured by percent change in NREM SWA from the first to last 20 minutes of sleep. After controlling for napping, age, sex, race, CDR, ApoE4 status, Aβ42/40, AHI, and PLMI in a regression model, sleep midpoint was associated with SWA dissipation (*p* = .002; (Table 7). Additionally, sleep midpoint was significantly correlated with dissipation (*r* = 0.231, *p* = .001) in a bivariate correlation analysis. Given that greater negative percent change in NREM SWA between the first and last 20 minutes of sleep indicates greater dissipation, these results indicate that an earlier sleep midpoint is associated with greater NREM SWA dissipation.

**Table 7.** Dissipation of NREM Slow-Wave Activity

	Change in 1–4.5 Hz NREM SWA activity from first to last 20 minutes of sleep	
Covariates	Estimate (β)	*P*-value
Sleep timing	0.0574	**.002**
Age	0.002	.654
Sex	0.010	.823
Race	0.105	.192
CDR	−0.004	.949
ApoE4 status	0.032	.538
Aβ42/40	−0.097	.930
AHI	0.004	.116
PLMI	0.0006	.497
Daytime minutes napping	0.001	.170

NREM, non-rapid-eye movement; SWA, slow-wave activity; TST, total sleep time; SOL, sleep onset latency; min, minutes; CDR, clinical dementia rating; ApoE4 status, apolipoprotein E ε4 positive; AHI, apnea–hypopnea index; PLMI, periodic limb movement index; Aβ, amyloid-beta.

Bold *P*-values are significant at <0.05.

### Actigraphy and self-reported sleep parameters

For sleep parameters measured by actigraphy, more awakenings and longer total sleep time were associated with later sleep midpoint (Table 8). Interestingly, higher CDR and AHI were both significantly associated with lower sleep efficiency, longer sleep onset latency, and/or a greater number of awakenings. Later sleep timing and higher CDR were associated with longer self-reported total sleep time (Table 9). An interaction between midpoint of sleep and CDR was not significant in any of the models (all *p* > .05, data not shown).

**Table 8.** Actigraphy Sleep Variables

	Dependent variables									
	TST (min)		Sleep efficiency (%)		SOL (min)		WASO (min)		Awakenings (#)	
Covariates	Estimate (β)	*P*-value	Estimate (β)	*P*-value	Estimate (β)	*P*-value	Estimate (β)	*P*-value	Estimate (β)	*P*-value
Sleep timing	6.289	**.041**	−0.700	.100	1.436	.118	1.641	.101	2.157	**.001**
Age	0.100	.892	−0.086	.393	0.084	.708	0.189	.429	0.201	.191
Sex	26.571	**<.001**	1.617	.111	−2.596	.237	9.315	**<.001**	−1.893	.219
Race	−39.000	**.004**	−3.297	.073	6.800	.087	−12.088	**.005**	−1.353	.626
CDR	−13.833	.152	−6.026	**<.001**	11.302	**<.001**	−1.378	.660	6.585	**.001**
ApoE4 status	10.879	.202	0.771	.513	−0.239	.925	3.935	.157	−1.126	.529
Aβ42/40	88.351	.629	9.440	.709	−57.531	.295	17.859	.764	−1.019	.979
AHI	−0.558	.156	−0.148	**.007**	0.127	.283	−0.243	.059	0.446	**<.001**
PLMI	−0.161	.295	−0.032	.133	0.064	.164	0.003	.960	−0.018	.587
Daytime minutes napping	−0.004	.981	0.009	.719	−0.007	.898	−0.041	.477	−0.068	.065

TST, total sleep time; SOL, sleep onset latency; WASO, wake after sleep onset; min, minutes; CDR, clinical dementia rating; ApoE4 status, apolipoprotein E ε4 positive; AHI, apnea–hypopnea index; PLMI, periodic limb movement index; Aβ, amyloid-beta.

Bold *P*-values are significant at <0.05.

**Table 9.** Self-Reported Sleep Variables

	Dependent variables					
	TST (min)		SOL (min)		Awakenings (#)	
Covariates	Estimate (β)	*P*-value	Estimate (β)	*P*-value	Estimate (β)	*P*-value
Sleep timing	14.636	**<.001**	−0.198	.869	−0.002	.974
Age	−0.545	.530	0.431	.136	0.004	.836
Sex	0.624	.943	12.371	**<.001**	0.218	.229
Race	−15.548	.324	−5.527	.294	−0.323	.322
CDR	25.940	**.024**	−2.835	.455	−0.031	.897
ApoE4 status	14.366	.157	2.414	.473	−0.072	.731
Aβ42/40	20.661	.924	116.868	.106	0.531	.908
AHI	0.005	.991	0.029	.857	0.010	.292
PLMI	−0.024	.897	−0.002	.969	−0.001	.843
Daytime minutes napping	−0.059	.778	0.134	.053	−0.002	.617

TST, total sleep time; SOL, sleep onset latency; min, minutes; CDR, clinical dementia rating; ApoE4 status, apolipoprotein E ε4 positive; AHI, apnea–hypopnea index; PLMI, periodic limb movement index; Aβ, amyloid-beta.

Bold *P*-values are significant at <0.05.

## Discussion

In this study, the later midpoint of sleep or sleep timing was associated with measures reflecting poorer sleep quality such as lower time in REM sleep and longer REM onset latency. This association, however, differed depending on the sleep measurement method used. Since a greater number of nighttime awakenings measured by actigraphy was associated with later midpoint of sleep, this suggests that individuals with later sleep timing may have more restless sleep. Similarly, only self-reported and actigraphy-measured total sleep time were related to later sleep timing; total sleep time measured by single-channel EEG was not significantly associated with the midpoint of sleep.

Interestingly, < 1 Hz and 1–2 Hz NREM SWA increased as the midpoint of sleep was delayed. This association was not observed at faster frequencies of 2–3 and 3–4 Hz, and remained significant even after adjusting for multiple potential factors that affect NREM SWA including age, sex, amyloid pathology (i.e. CSF Aβ42/40), and self-reported minutes napping during the day. Since individuals with later sleep timing had evidence of poorer sleep quality (e.g. more nighttime awakenings, longer REM onset latency), we hypothesize that higher NREM SWA may represent a homeostatic response to decreased sleep quality. Daytime napping, a marker of daytime sleep need, was also associated with increased < 1 Hz and 1–2 Hz NREM SWA. However, we found that an earlier sleep midpoint was associated with a greater percent change in NREM SWA (i.e. greater dissipation of sleep pressure). Previous work reported that the decrease in NREM SWA during sleep was significantly greater in healthy young adults with morning types [56, 57]. In older adults, a decreased NREM SWA response to high sleep pressure was observed only in the frontal brain regions as measured by the single-channel EEG device used in this study [58]. These findings strongly suggest that the sleep homeostatic response differs with chronotype and may be due to age-related changes in the circadian timing system. Further investigations of the associations between circadian mechanisms and sleep homeostasis are needed in older adults at risk for AD.

Individuals with a self-reported black ethnic background have been reported to have earlier chronotypes than individuals of self-reported white ethnic background [59] and African Americans have been reported to have a shorter circadian period than European Americans [60]. For example, a study from the UK Biobank cohort found that a matched sample (*N* = 2044; 50% self-reported black ethnic background) with a mean age of 52 had a significantly greater prevalence of self-reported morning types based on a single-question survey item in participants with self-reported black ethnic background [59]. In contrast, participants with self-reported black ethnic background in our cohort had later sleep midpoints. This direction of effect has been reported before. In a study with 578 African American and 823 white individuals, actigraphic mean weekday midsleep time was 27 minutes later in African Americans than in whites (*p* = .02), and weekend midsleep time was 20 minutes later (*p* = .03) [61]. That study had a mean age of 68, and also discusses the possibility of older age accounting for the difference in results from prior studies. Since it is known that the midpoint of sleep shifts earlier throughout adulthood [62], future research is needed to assess if African Americans have a faster transition to early sleep midpoint and if this is modified by age. Racial differences in chronotype are likely attributable to social determinants of health and future studies are also needed to assess their role.

The current finding contrary to the UK Biobank and other findings could be due to the comparatively small sample size (9.9% African American in the current cohort), the older age of participants in the present study (mean age = 74), differences in geographical location, differences in factors included in each study’s respective models (i.e. AD biomarkers such as CSF Aβ42/40), or methodological differences in measuring the midpoint of sleep or chronotype. The participants in the regression analyses may not be representative of the general population, with an educational attainment of 15.3 years among African Americans and 16.5 years among non-African Americans. Although only 24 African American participants completed a lumbar puncture and were included in the fully adjusted models, 58 participants had data available to calculate the midpoint of sleep and the same relationship was observed without adjusting for other factors.

These results indicate that midpoint of sleep or sleep timing is associated with measures indicating poorer sleep quality. Since we calculated the midpoint of sleep based on each individual’s sleep behavior, we propose that this may be modified as part of a treatment plan to improve sleep quality. Chronotype can be modified by light exposure [63]; for example, exposure to 9500 lux resulted in a shift of 4.5 hours earlier in young men [64]. Melatonin administration resulted in a shift in sleep timing of around 1.5 hours earlier [65]. Among participants with delayed sleep phase syndrome, an attempt to avoid both evening light exposure and napping, while attempting to advance sleep time resulted in a shift in sleep timing of about 1 hour earlier in the absence of bright light therapy [66].

CDR was significantly associated with multiple sleep variables although there were differences depending on the method. For instance, higher CDR (i.e. greater cognitive impairment) was associated with longer REM latency, but not other sleep parameters measured by single-channel EEG. Higher CDR was also associated with lower sleep efficiency, longer sleep onset latency, and a greater number of nighttime awakenings measured by actigraphy, and longer self-reported total sleep time. With each of these variables, later sleep timing and mild cognitive impairment were either both positively or both negatively associated with different sleep parameters in the linear models. That is, the same directions of effects were observed, indicating the potential for sleep midpoint to be useful as a covariate in certain models.

Future research is needed to determine if behavioral sleep interventions targeting the timing of sleep improve sleep quality and potentially cognitive performance. For example, will normalizing very late sleep timing improve sleep parameters such as REM sleep or decreased intra-night sleep–wake time variability? Sleep timing may be a partial confound of an association of a sleep measure with an outcome (e.g. AD pathology or cognitive deficit), which if not accounted for, may lead to spurious associations. Future studies are needed to test the effect of behavioral interventions on the midpoint of sleep to improve sleep quality in older adults and as a factor that needs to be accounted for if using sleep measures as markers of AD risk. This could be accomplished through behavioral recommendations and education about the effects of abnormal sleep phase, bright light therapy beginning in the morning, limiting light exposure in the evening, modifying other zeitgebers such as exercise and meal timing, melatonin, or a combination thereof. Further research can also elucidate how inter-night variability in sleep timing affects sleep quality and dementia, the interaction between chronotype and cognitive function in sleep outcomes, and the longitudinal association between sleep timing and AD biomarkers.

This study provides a detailed characterization of the association of the midpoint of sleep or sleep timing with a range of sleep parameters in older adults with and without symptomatic AD. Results indicate that a later midpoint of sleep is associated with different sleep parameters even after adjustment for potential confounding factors. Several of the sleep measures suggest that worse sleep quality (e.g. decreased time in REM sleep) is associated with later sleep timing. However, the relationship between other sleep parameters is unclear and warrants further investigation. For instance, later sleep timing and daytime napping are associated with increased NREM SWA. However, later sleep midpoint was associated with less dissipation of NREM SWA. NREM SWA has been proposed as a marker of brain function in individuals at risk for AD. Further research is needed to establish the role of sleep timing on NREM SWA and other sleep measures, how sleep timing should be taken into account when assessing the effect of sleep on AD, and to assess the effect of the midpoint of sleep on the homeostatic response to increased sleep pressure in older adults.

### Limitations

This study has several strengths, including a large sample size, actigraphic, EEG, and self-report sleep measures, and AD biomarker data from CSF. It does, however, have limitations. Due to the cross-sectional study design, we cannot use the temporality of measures to infer the direction of potential causal associations. We also adjusted for sleep apnea and periodic leg movements during sleep using AHI and PLMI measured by an HSAT and calculated by monitoring time rather than sleep time; this may have resulted in a biased finding in participants with lower sleep efficiency. In addition, we did not correct for multiple comparisons, so future research replicating our findings will be valuable.

## Supplementary Material

zpae023_suppl_Supplementary_Figure_S1
