# Supplementary material for: Midpoint of sleep is associated with sleep quality in older adults with and without symptomatic Alzheimer’s disease
Source: Sleep Adv. 2024 Apr 15;5(1):zpae023. doi: 10.1093/sleepadvances/zpae023 (PMC11071685; doi:10.1093/sleepadvances/zpae023)
Supplement: zpae023_suppl_Supplementary_Figure_S1 [file zpae023_suppl_supplementary_figure_s1.docx]

**SUPPLEMENTARY MATERIALS**

**Midpoint of Sleep is Associated with Sleep Quality in Older Adults with and without Symptomatic Alzheimer’s Disease**

Scott C. Sauers;^1^ Cristina D. Toedebusch;^1^ Rachel Richardson;^1^ Adam P. Spira;^2,3,4^ John C. Morris;^1,5^ David M. Holtzman;^1,5,6,7^ Brendan P. Lucey^1,6,7*^

^1^Department of Neurology, Washington University School of Medicine, St Louis, MO 63110

^2^Department of Mental Health, The Johns Hopkins Bloomberg School of Public Health, Baltimore, MD

^3^Department of Psychiatry and Behavioral Sciences, The Johns Hopkins School of Medicine, Baltimore, MD 21205

^4^The Johns Hopkins Center on Aging and Health, Baltimore, MD 21205

^5^Knight Alzheimer Disease Research Center, Washington University School of Medicine, St Louis, MO 63110

^6^Center on Biological Rhythms and Sleep, Washington University School of Medicine, St Louis, MO 63110

^7^Hope Center for Neurological Disorders, Washington University School of Medicine, St Louis, MO 63110

*corresponding author

Brendan P. Lucey, MD, MSCI

Washington University School of Medicine

Campus Box 8111

660 South Euclid Avenue

St Louis, MO 63110

Phone: 314-747-3805

Fax: 314-747-3813

Email: [luceyb@wustl.edu](mailto:luceyb@wustl.edu)


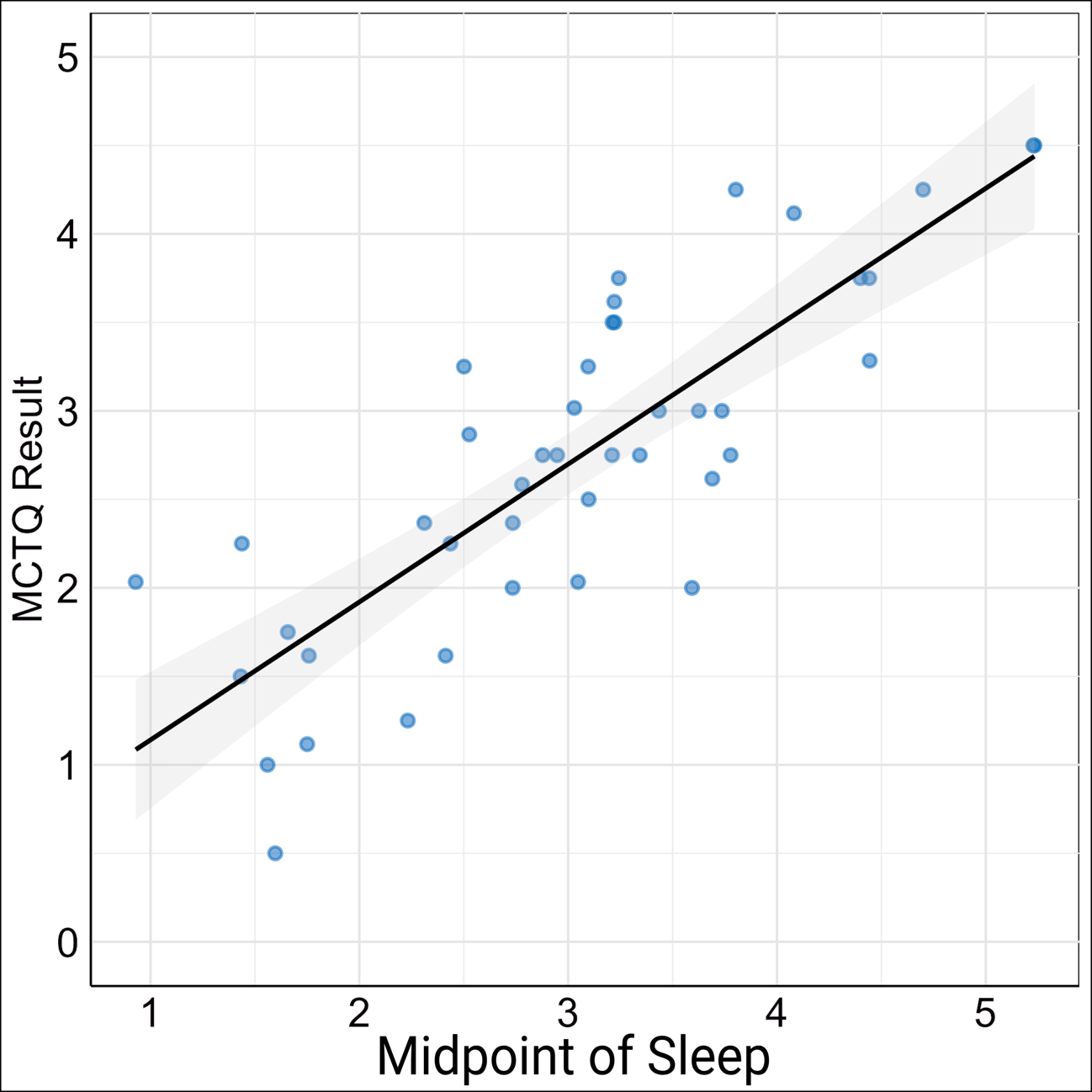


Supplementary Figure 1: Relationship of midpoint of sleep and Munich Chronotype Questionnaire. A highly significant correlation was observed between the midpoint of sleep and the Munich Chronotype Questionnaire (MCTQ) (r=0.823; p=1.278x10^-11^).
